# Supplementary material for: Lipid composition-driven sorting of ABHD5 between monolayer and bilayer surfaces
Source: J Lipid Res. 2026 Oct 7;67(11):101166. doi: 10.1016/j.jlr.2026.101166 (PMC13756748; doi:10.1016/j.jlr.2026.101166)
Supplement: Supplementary file 1 [file mmc1.pdf]

Table S1. Pairwise comparison *p*-values for monolayer, bilayer, and sorting values across neutral lipid types (**Fig. 2**).

**BILAYER vs BILAYER comparisons:**		
Pairs	*P* values	Summary
DO vs 50:50	2.236e-31	****
50:50 vs TO	3.240e-07	****
DO vs TO	4.146e-32	****
**MONOLAYER vs MONOLAYER comparisons:**		
Pairs	*P* values	Summary
DO vs 50:50	1.617e-23	****
50:50 vs TO	0.0019	**
DO vs TO	4.007e-18	****
**SORTING PARTITION COEFFICIENT comparisons:**		
Pairs	*P* values	Summary
DO vs 50:50	7.663e-06	****
50:50 vs TO	4.441e-09	****
DO vs TO	1.041e-09	****

Table S2. Pairwise comparison *p*-values for LUVs and GUVs with varying neutral lipid composition and concentrations (**Fig. 4**)

**LUV Pairs**	***P* values**	**Summary**
0% NL vs 1% TO	4.641e-12	****
0% NL vs 1% CE	0.238	n.s.
0% NL vs 5% CE	6.5411e-06	****
0% NL vs 25% CE	0.0013	**
0% NL vs 1% DO	0.265	n.s.
0% NL vs 5% DO	5.385e-09	****
0% NL vs 25% DO	3.682e-35	****
5% DO vs 25% DO	7.405e-22	****
5% CE vs 25% CE	8.719e-17	****

**GUV Pairs**	***P* values**	**Summary**
0% NL vs 2.5% TO	0.996	n.s.
0% NL vs 5% CE	0.0002457	***
0% NL vs 10% CE	0.187	n.s.
0% NL vs 25% CE	4.827e-06	****
0% NL vs 5% DO	6.403e-28	****
0% NL vs 10% DO	2.403e-41	****
0% NL vs 25% DO	5.898e-15	****
5% DO vs 25% DO	0.002516	**
5% CE vs 25% CE	0.994	n.s.

Table S3. Pairwise comparison *p*-values for monolayer, bilayer, and sorting coefficient measurements across phospholipid conditions for TO DEV (**Fig. 5A, B**).

**BILAYER vs BILAYER comparisons:**		
Pairs	*P* values	Summary
PC vs PC:PE	0.835	n.s.
PC vs PC: PE: PI	0.471	n.s.
PC vs PC: PE: PS	0.461	n.s.
PC: PE vs PC: PE: PI	0.125	n.s.
PC: PE vs PC: PE: PS	0.363	n.s.
PC: PE: PI vs PC: PE: PS	0.002	**
**MONOLAYER vs MONOLAYER comparisons:**		
Pairs	*P* values	Summary
PC vs PC:PE	9.475e-05	****
PC vs PC: PE: PI	4.528e-06	****
PC vs PC: PE: PS	5.990e-05	****
PC: PE vs PC: PE: PI	0.864	n.s.
PC: PE vs PC: PE: PS	0.678	n.s.
PC: PE: PI vs PC: PE: PS	0.491	n.s.
**SORTING PARTITION COEFFICIENT comparisons:**		
Pairs	*P* values	Summary
PC vs PC:PE	0.583	n.s.
PC vs PC: PE: PI	0.643	n.s.
PC vs PC: PE: PS	0.941	n.s.
PC: PE vs PC: PE: PI	0.446	n.s.
PC: PE vs PC: PE: PS	0.657	n.s.
PC: PE: PI vs PC: PE: PS	0.733	n.s.

Table S4. Pairwise comparison *p*-values for monolayer, bilayer, and sorting coefficient measurements across phospholipid conditions for 50:50 v/v TO:DO DEV (**Fig. 5C, D**).

**BILAYER vs BILAYER comparisons:**		
Pairs	*P* values	Summary
PC vs PC:PE	0.773	n.s.
PC vs PC: PE: PI	0.601	n.s.
PC vs PC: PE: PS	0.169	n.s.
PC: PE vs PC: PE: PI	0.769	n.s.
PC: PE vs PC: PE: PS	0.249	n.s.
PC: PE: PI vs PC: PE: PS	0.487	n.s.
**MONOLAYER vs MONOLAYER comparisons:**		
Pairs	*P* values	Summary
PC vs PC:PE	0.269	n.s.
PC vs PC: PE: PI	0.035	*
PC vs PC: PE: PS	0.164	n.s.
PC: PE vs PC: PE: PI	0.161	n.s.
PC: PE vs PC: PE: PS	0.676	n.s.
PC: PE: PI vs PC: PE: PS	0.311	n.s.
**SORTING PARTITION COEFFICIENT comparisons:**		
Pairs	*P* values	Summary
PC vs PC:PE	5.938e-03	**
PC vs PC: PE: PI	0.074	n.s.
PC vs PC: PE: PS	0.115	n.s.
PC: PE vs PC: PE: PI	3.866e-02	*
PC: PE vs PC: PE: PS	0.053	n.s.
PC: PE: PI vs PC: PE: PS	0.821	n.s.
