# Supplementary material for: Lipid composition-driven sorting of ABHD5 between monolayer and bilayer surfaces
Source: J Lipid Res. 2026 Oct 7;67(11):101166. doi: 10.1016/j.jlr.2026.101166 (PMC13756748; doi:10.1016/j.jlr.2026.101166)
Supplement: Supplementary file 2 [file mmc2.pdf]

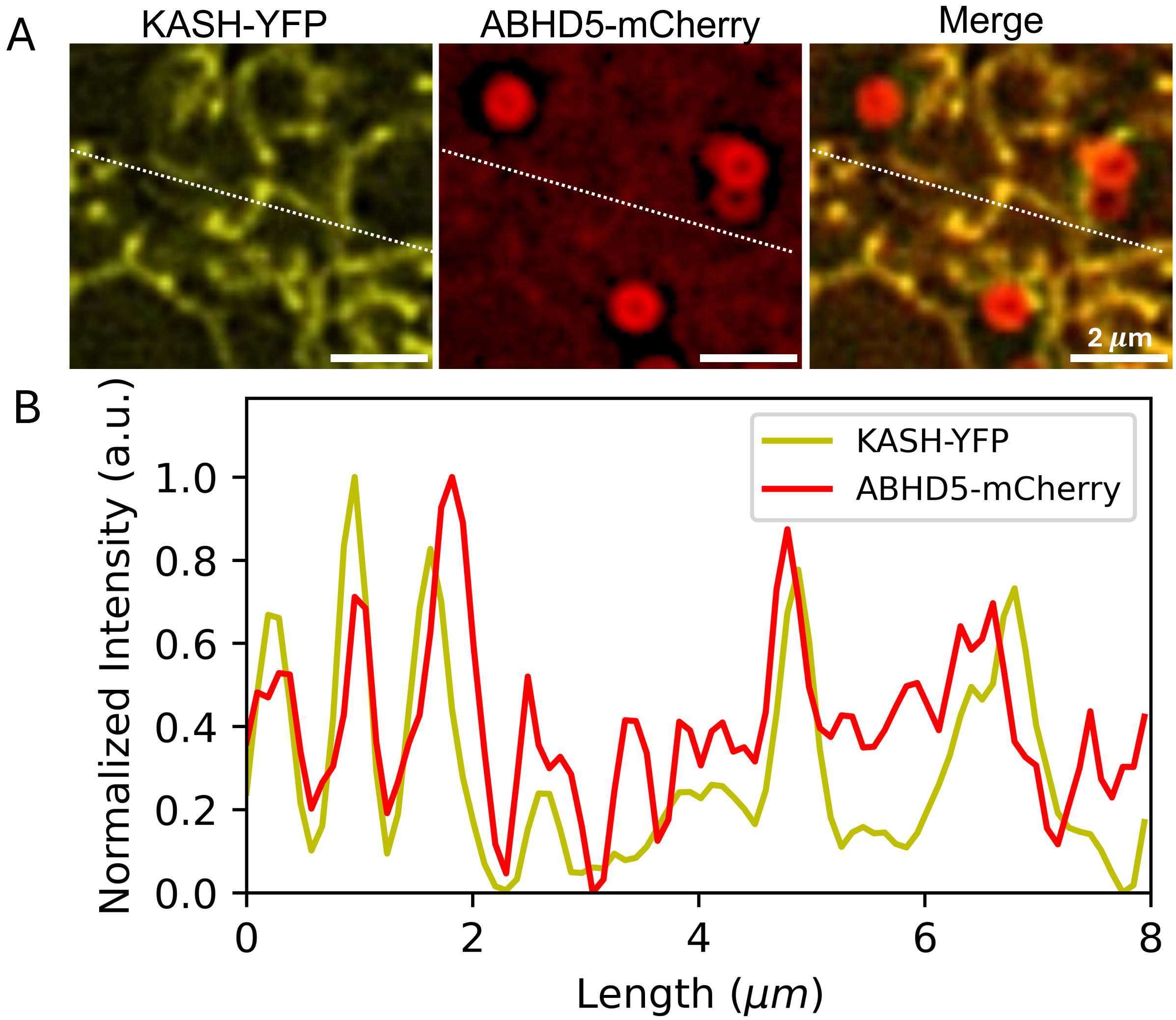

Figure S1: ABHD5 localizes to LDs and ER. (A) Enlarged view of the region indicated in the Figure 1A (right). Fluorescent image showing KASH-YFP (left), ABHD5-mCherry (center), and the merged channels (right). KASH-YFP labels the ER network, while ABHD5-mCherry is enriched on LDs and shows signal associated with the ER. The white dashed line indicates the region used for line-scan analysis. (B) Normalized fluorescence intensity profiles of KASH-YFP and ABHD5-mCherry along the indicated line scan. Overlapping peaks in KASH-YFP and ABHD5-mCherry fluorescence indicate spatial association of ABHD5 with the ER.

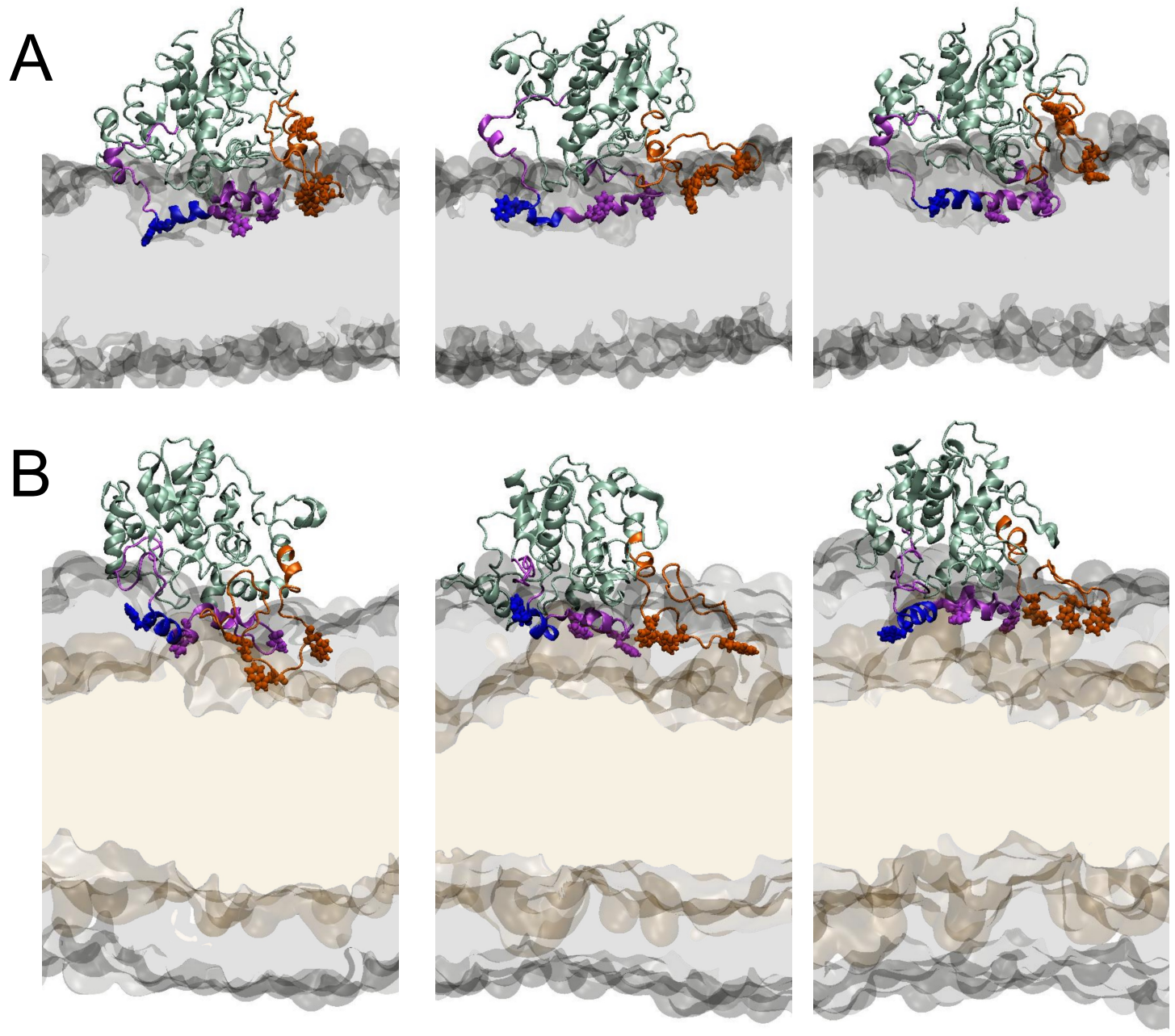

Figure S2: All-atom molecular dynamics snapshots of ABHD5 binding to (A) a phospholipid bilayer or (B) phospholipid monolayers surrounding a layer of triolein. The PC, PE, and PI phospholipids (*gray*) and neutral lipids (*brown*) attract the ABHD5 N-terminal region (aa1-43, *orange*) and the insertion domain (aa180-230, *purple*), which includes the lid (aa192-207, *blue*). W21, W25, W29, S199, F210, and F222 are bulky hydrophobic residues instrumental in lipid binding (*space-filling*). One view from each of the three replicas of each simulation is shown.

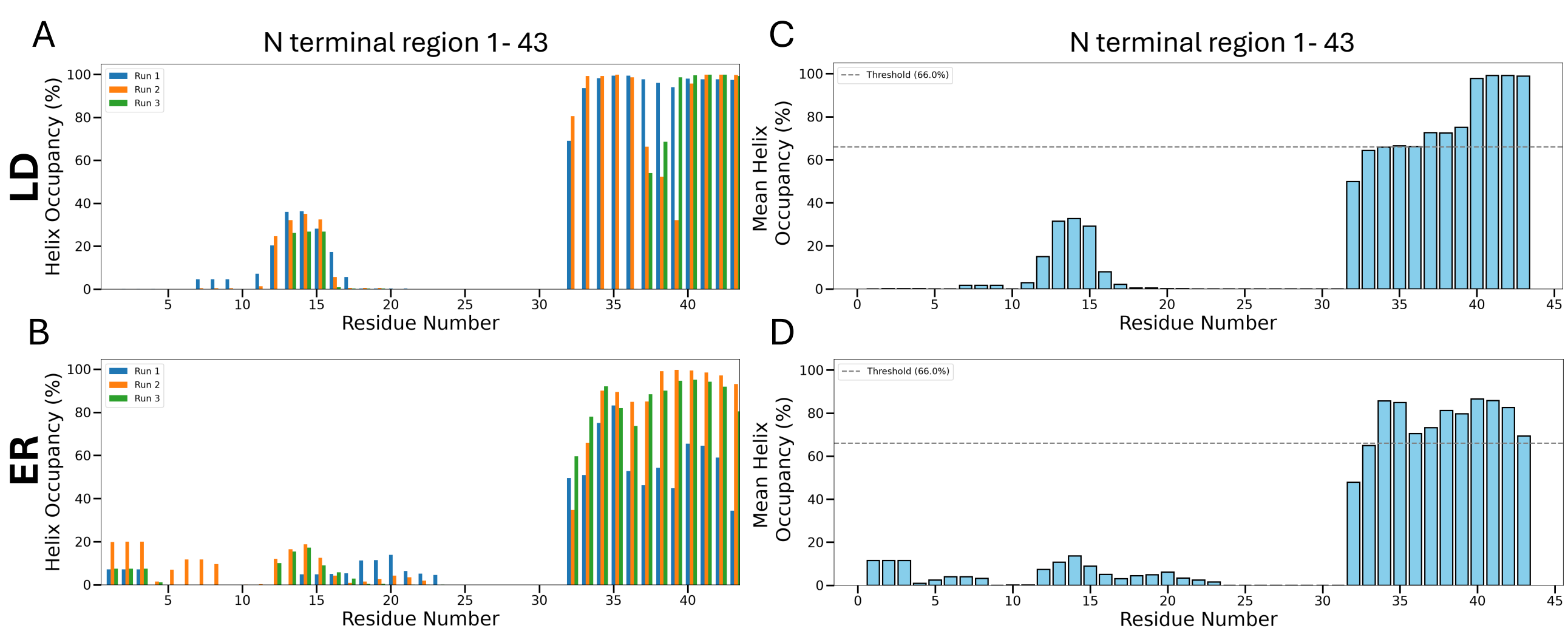

Figure S3: Helix occupancy was calculated for residues from the three repeated all-atom molecular dynamics simulations in the membrane binding regions 1- 43.

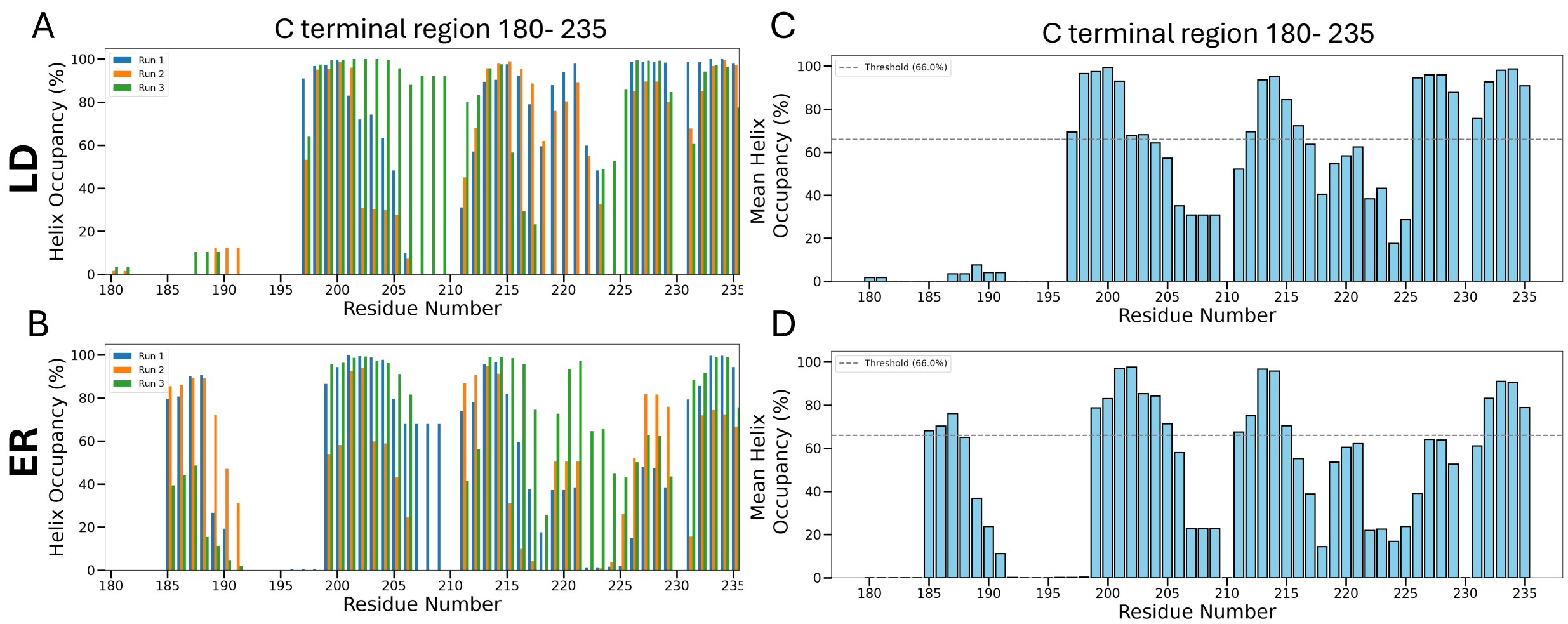

Figure S4. Helix occupancy was calculated for residues from the three repeated all-atom molecular dynamics in the membrane binding regions 180- 235.

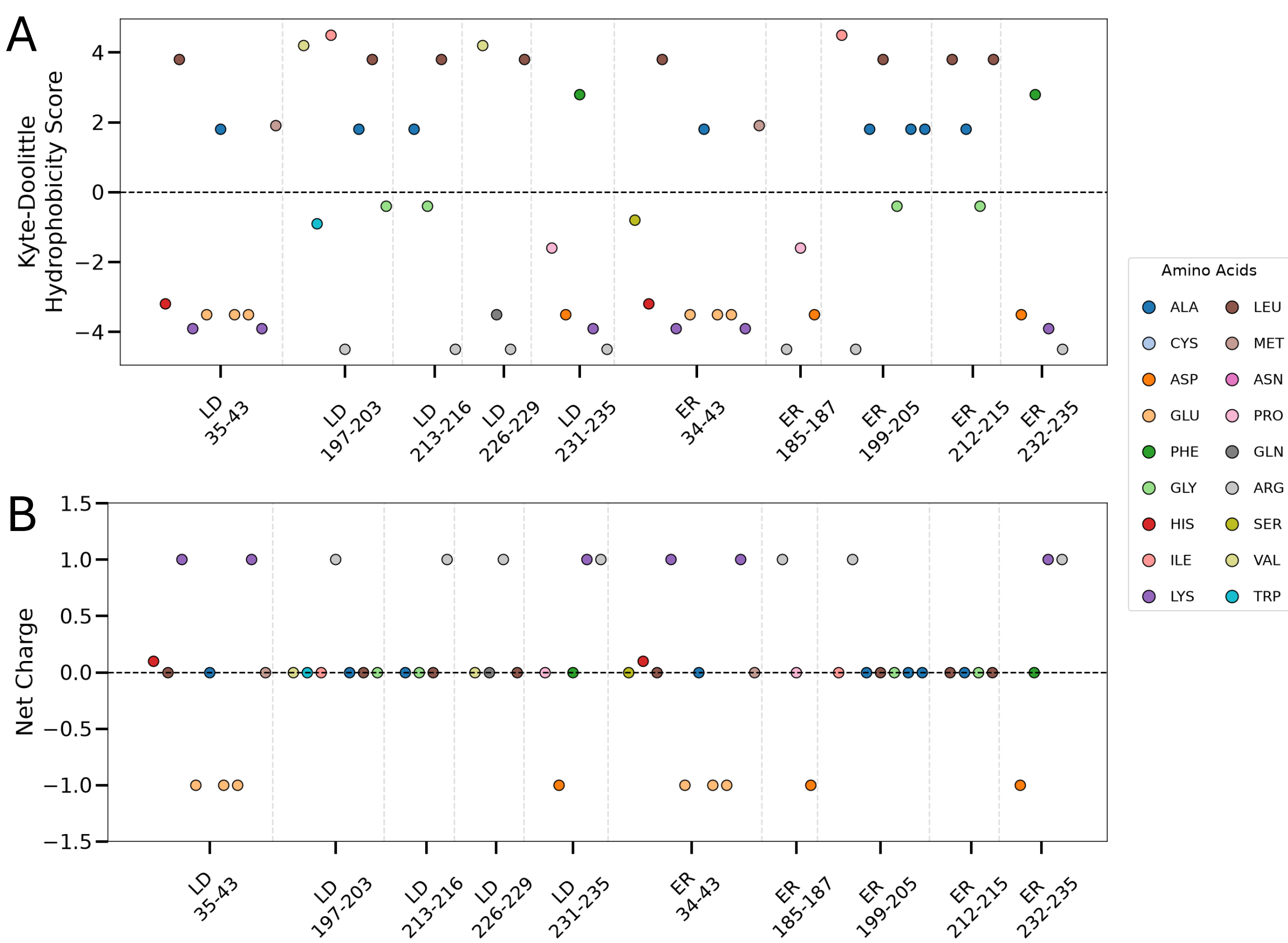

Figure S5: Physicochemical properties of persistent helical motifs support lipid-dependent membrane binding

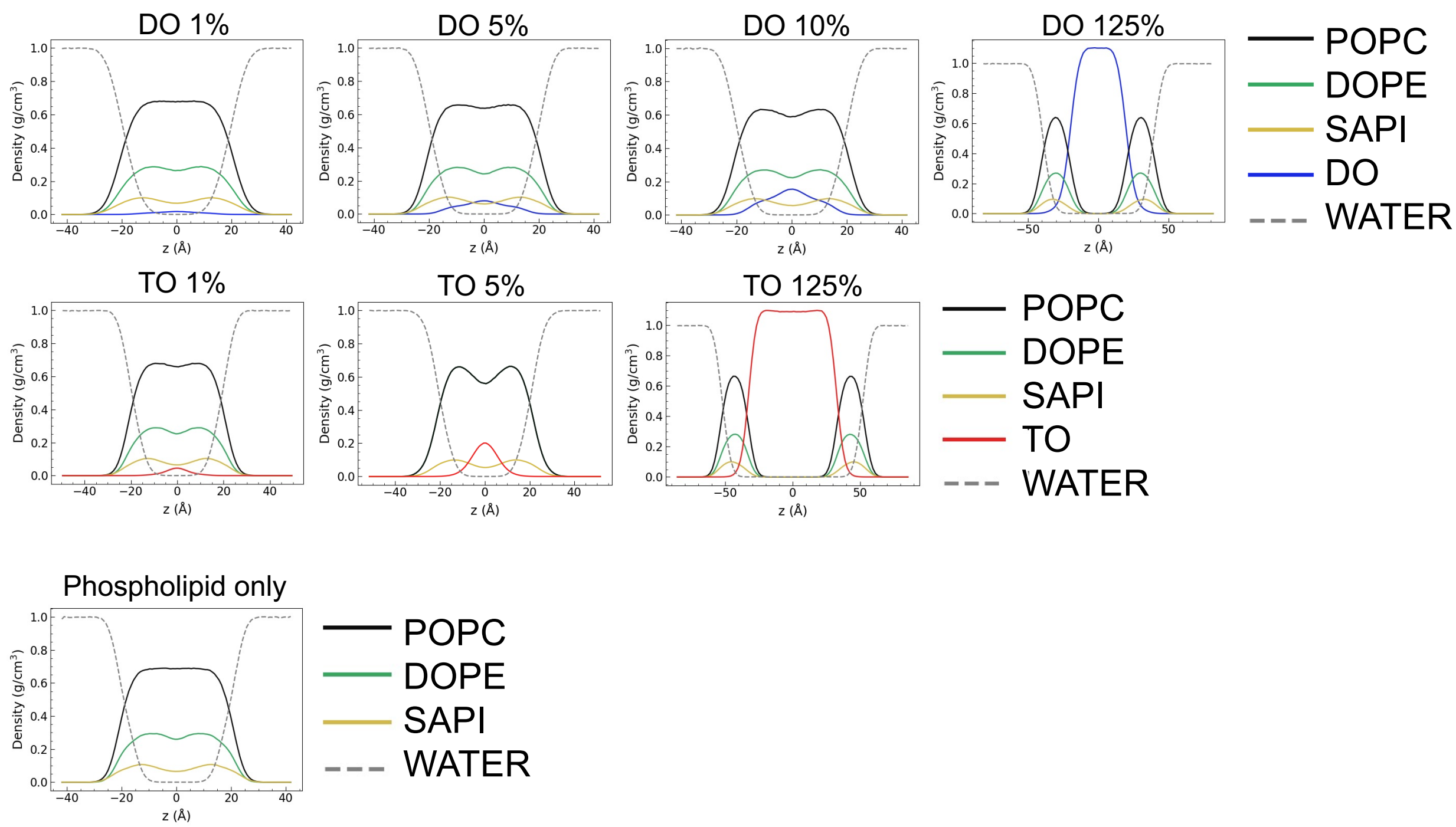

Figure S6: Z density profile for phospholipid and neutral lipids at varying concentrations.
